# Restructuring of contralateral gray matter volume associated with cognition in patients with unilateral temporal lobe glioma before and after surgery

**DOI:** 10.1002/hbm.24911

**Published:** 2019-12-28

**Authors:** Guanjie Hu, Xinhua Hu, Kun Yang, Dongming Liu, Chen Xue, Yong Liu, Chaoyong Xiao, Yuanjie Zou, Hongyi Liu, Jiu Chen

**Affiliations:** ^1^ Department of Neurosurgery The Affiliated Brain Hospital of Nanjing Medical University Nanjing Jiangsu China; ^2^ Institute of Brain Functional Imaging Nanjing Medical University Nanjing Jiangsu China; ^3^ Department of Radiology The Affiliated Brain Hospital of Nanjing Medical University Nanjing Jiangsu China; ^4^ Institute of Neuropsychiatry The Affiliated Brain Hospital of Nanjing Medical University, Fourth Clinical College of Nanjing Medical University Nanjing Jiangsu China

**Keywords:** cognition, gray matter volume, temporal lobe glioma, voxel‐based morphometry

## Abstract

Glioma can cause variable alterations to the structure and function of the brain. However, there is a paucity of studies on the gray matter (GM) volume alterations in the brain region opposite the temporal glioma before and after surgery. Therefore, the present study was initiated to investigate the alternation in contralateral homotopic GM volume in patients with unilateral temporal lobe glioma and further, assess the relationship between GM volume alternations with cognition. Eight left temporal lobe glioma patients (LTPs), nine right temporal lobe glioma patients (RTPs), and 28 demographically matched healthy controls (HCs) were included. Using voxel‐based morphometry method, alternations in the contralateral homotopic GM volume in patients with unilateral temporal lobe glioma was determined. Furthermore, the correlation analysis was performed to explore the relationship between cognitive function and altered GM volume. In the preoperative analysis, compared to HCs, LTPs exhibited increased GM volume in right inferior temporal gyrus and right temporal pole (superior temporal gyrus), and, RTPs presented increased GM volume in left inferior temporal gyrus. In the postoperative analysis, compared to HCs, RTPs presented increased GM volume in left middle temporal gyrus. Furthermore, the increased GM volume was significantly positively correlated with the memory test but negatively correlated with the visuospatial test. This study preliminarily confirmed that there were compensatory changes in the GM volume in the contralateral temporal lobe in unilateral temporal lobe glioma patients. Furthermore, alterations of GM volume may be a mechanism for cognitive function compensation.

## 1 INTRODUCTION

Glioma constitutes over 70% of primary malignant tumor of the central nervous system (CNS) (Gusyatiner & Hegi, 2018). Glioblastoma remains the most aggressive and highly invasive type of glioma, representing approximately 14.9% of all CNS tumors and 46.6% of all primary malignant brain tumors (Ostrom et al., 2016). Currently, surgical resection, followed by radiation therapy and adjuvant chemotherapy is the standard of care for patients with glioma (Duffau & Taillandier, 2015); however, in spite of these treatments, 5‐year survival rates (5%) remain poor (Ghinda, Wu, Duncan, & Northoff, 2018). To our knowledge, focal deficits caused by glioma are not restricted to the area where the tumor is located and where it has caused local damage; however, it may impact the global dysfunctioning of the brain (Almairac, Duffau, & Herbet, 2018; Hart, Romero‐Garcia, Price, & Suckling, 2019; Maesawa et al., 2015). Some studies have suggested that localized tumors can affect the function of distant part of the brain (Liu et al., 2019; Park, Kim, Kim, Kim, & Shim, 2015; Zhang et al., 2016). Indeed, structural changes in the regions distant from brain tumor have also been confirmed by some studies (Almairac et al., 2018; Bouwen et al., 2018). These structural changes have been correlated with cognition decline (Bouwen et al., 2018), though cognitive functions are not always caused by the alternation in the brain, sometimes the cognitive function recovers over time (Voytek et al., 2010), which may be attributed to the brain plasticity. Therefore, by exploring the structural alterations contributing to the integrity of cognitive function, may help to improve the preoperative plan and may result in the development of more rational strategie7s to facilitate neurorehabilitation of patients with glioma.

Furthermore, it has been reported that the eloquent brain areas in patients with glioma were frequently infiltrated by tumor; however, may not result in detectable neurological deficits due to the plasticity of the CNS (Almairac et al., 2018; Duffau, 2014). Accumulating studies have reported that the plasticity is caused by the functional or morphological remodeling of the neural organization of CNS (Duffau, 2005; Duffau, 2006) and may present at four at different levels: (a) functional remodeling occurring within the tumor; (b) functional remodeling occurring around the tumor; (c) recruitments in remote areas within the lesion hemisphere; (d) recruitments in areas within the contralateral hemisphere. These forms of functional redistribution can occur alone or together (Duffau, 2014). And all these distributions may be associated with the changes between synapses (von Bernhardi, Bernhardi, & Eugenin, 2017), including changes in the circuit connectivity involving formation, removal, or enlargement of synapses (Fauth & Tetzlaff, 2016). Indeed, synaptic plasticity may occur without accompanied by changes in synaptic number, division, density, or total area, owing to the changes in electronic properties of dendrites (von Bernhardi et al., 2017). Through the ability of brain plasticity, function of patients with glioma may not decline rapidly due to tumor invasion, which may be attributed to mechanisms compensated for the tumor‐induced functional or morphological remodeling in patients with glioma.

Voxel‐based morphometry (VBM) is a widely used automated technique for the analysis of neuroanatomical images, using that allows investigation of focal differences in brain anatomy, using the statistical approach of parametric mapping (Ashburner & Friston, 2000; Vanasse et al., 2018). Moreover, VBM simply involves a voxel‐wise comparison of the local concentration or volume of gray matter (GM) and white matter (WM) between subject groups. And the VBM method does not favor a particular structure but can assess anatomical differences across the whole brain (Matsuda, 2016). This method has been successfully used to detect structural changes of cerebral GM and WM in patients with Alzheimer's disease, acute focal cerebral infarction and depression (Ashburner & Friston, 2000; Dang et al., 2013; Liu et al., 2014; Matsuda, 2016). Furthermore, Almairac et al. found that patients with unilateral insular low‐grade glioma exhibited noticeable increase in GM volume only in the contralateral insular lobe (Almairac et al., 2018), indicating a plastic compensatory mechanism in the distant brain regions. Conceivably, these changes in human brain may be related to cognitive changes in patients with glioma (Derks, Reijneveld, & Douw, 2014). Besides, the temporal lobe is related to variety of cognitive functions, including the memory, verbal learning, language abilities, executive function, visuospatial memory, and so on (Kane, 2015; Noll et al., 2015; Noll, Bradshaw, Weinberg, & Wefel, 2017; Noll, Ziu, Weinberg, & Wefel, 2016). Therefore, the neurocognitive function may be affected by the damaged temporal lobe (Wu et al., 2011). However, structural changes in the contralateral homotopic in patients with unilateral temporal glioma remain elusive. Furthermore, it remains unknown whether structural changes can lead to cognition alteration. Meanwhile, we can minimize the influence of tumor infiltration and edema by investigating the changes of contralateral GM structure in patients with unilateral temporal lobe glioma.

Therefore, the present study was initiated to investigate the alteration in contralateral GM volume before and after surgery, and the relationship between the alterations in contralateral GM volume with cognitive function in unilateral temporal lobe glioma patients. We hypothesized that contralateral GM volume compensates for damaged glioma and the compensatory brain structure may be a structural basis of cognitive function compensation (Almairac et al., 2018; Bouwen et al., 2018; Duffau, 2005; Duffau, 2006).

## 2 METHODS

### 2.1 Participants

Seventeen patients with temporal glioma including eight patients with left temporal glioma (mean age 57.25 ± 7.52) and nine patients with glioma in right temporal lobe (mean age 51.56 ± 17.56) were enrolled from the Department of Neurosurgery in Affiliated Brain Hospital of Nanjing Medical University, Jiangsu province, China. Patients inclusion criteria were as follows: (a) histopathologically confirmed unilateral temporal glioma according to the 2007 World Health Organization (WHO) classification of Tumors of the CNS (Louis et al., 2016); (b) no self‐reported history of drug or alcohol abuse or substance abuse, no history of head injury, (c) no contraindications for participating in an magnetic resonance imaging (MRI) study, and (d) no neuropsychiatric illness and no illicit drug use over the past month. Due to the noncooperation and data loss of the patients, we only have nine patients postoperative MRI data, including six patients with left temporal glioma and three patients with right temporal glioma. Moreover, 28 demographically matched healthy controls (HCs; mean age 56.82 ± 7.74) with no brain disorders or diseases were also included in this study. To ensure these individuals represented a healthy comparison group, HCs were evaluated using unstructured clinical interviews to exclude individuals who had a history of severe systemic disease, or head trauma or psychological disorder. All of the patients and HCs were native Chinese descent and right‐handed according to the Edinburgh Handedness Inventory. This study was approved by the Institutional Ethical Committee for Clinical Research of the Affiliated Brain Hospital of Nanjing Medical University. Written informed consent was obtained from all participants. The data of subjects are not publicly available due to privacy or ethical restrictions.

### 2.2 MRI examination

All MRI images were acquired in 3.0 Tesla Verio Siemens scanner equipped with an eight‐channel phased‐array head radiofrequency coil in the Department of Radiology, Affiliated Brain Hospital of Nanjing Medical University. The structural scans were axially acquired using the high‐resolution three‐dimensional (3D) T1‐weighted magnetization‐prepared rapid gradient echo with the following acquisition parameters: repetition time (TR) = 1.9 s, echo time (TE) = 2.49 ms, time inversion (TI) = 900 ms, matrix = 256 × 256, flip angle (FA) = 9, slice thickness = 1 mm, and gap = 0.5 mm, and slice number = 176 slices covering the whole brain.

### 2.3 Neurocognitive assessments

All subjects included in this study were provided with unstructured clinical interview‐based neurocognitive assessments which were performed by two experienced neuropsychologists to ensure the reliability of the results. Several classical neurocognitive tests including digit span test (DST), memory test, visuospatial test, math exam test, digital symbol substitution test (DSST), mapping test, and similarity test.

### 2.4 Image preprocessing

All images were reviewed by a neuroradiologist for identifying artifacts during image acquisition and the presence of silent gross brain alterations. The 3D T1‐weighted images were preprocessed for VBM analyses using the Data Processing and Analysis of Brain Imaging (Yan, Wang, Zuo, & Zang, 2016) based on Statistical Parametric Mapping (SPM) program, version 8 (SPM8; SPM, http://www.fifil.ion.ucl.ac.uk/spm) implemented in MATLAB2014a (http://www.mathworks.com/products/matlab/) with the following preprocessing steps: first, we manually selected the anterior commissure as the origin (coordinate 0,0,0); next, each image were segmented into GM, WM, and cerebrospinal fluid (CSF) using SPM8. The Diffeomorphic Anatomical Registration Through Exponentiated Lie Algebra algorithm was then used to spatially normalize the segmented images (Ashburner & Friston, 2009). The segmented GM, WM, CSF maps were used to compute total intracranial volumes (TIVs) across all subjects (using in‐house code running in MATLAB); then, these images were spatially normalized, then these fully normalized images were resliced through trilinear interpolation to a final voxel size of 1.5 × 1.5 × 1.5 mm^3^ in Montreal Neurological Institute; an additional “modulation” step of multiplying each spatially normalized GM and WM image with its relative volume before and after normalization was also performed; finally, the resulting GM and WM images were smoothed using 8‐mm full width at half‐maximum Gaussian smoothing. The generated smoothened GM images were subjected to the following statistical analyses.

### 2.5 Voxel‐wise statistical analyses

**Figure 1:**
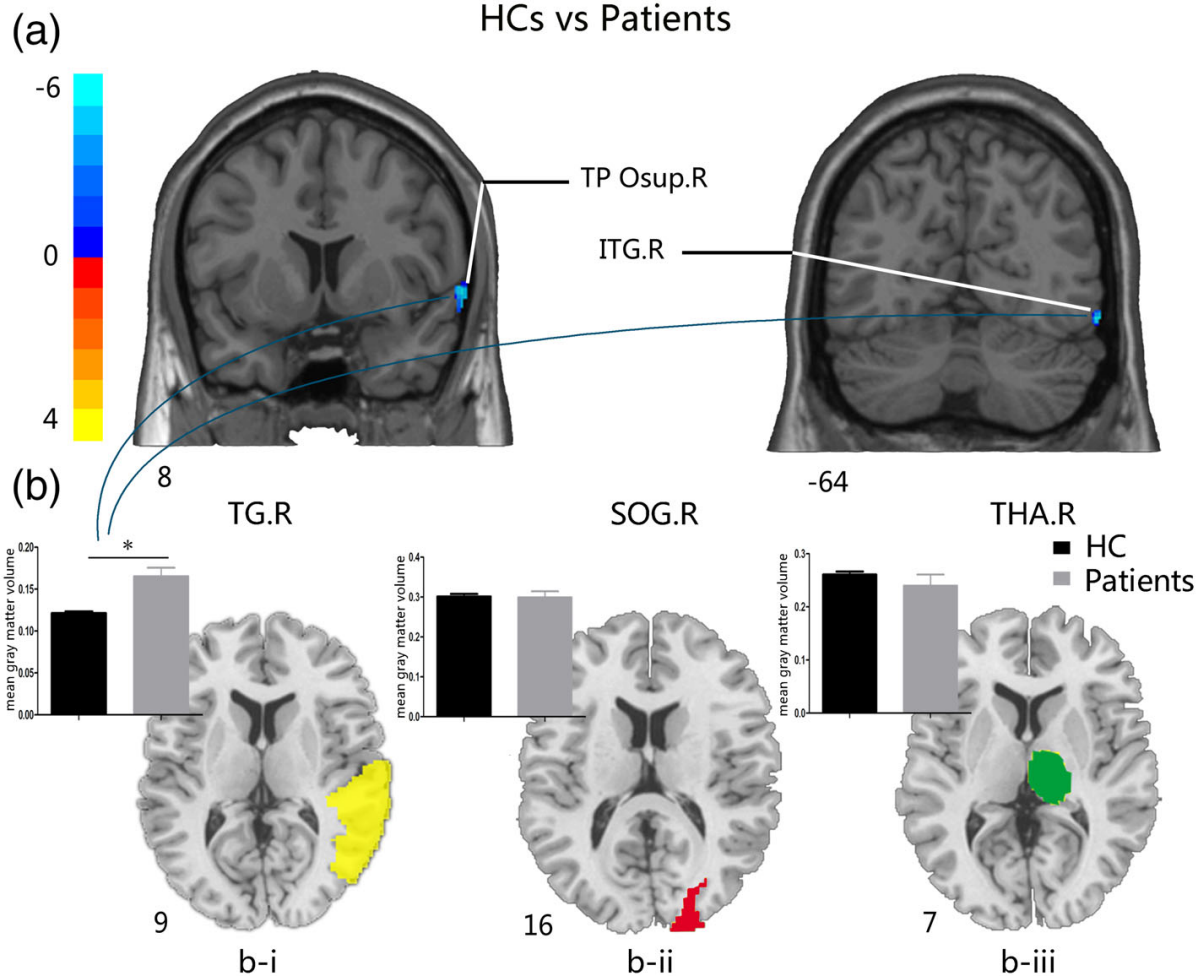
Comparisons of GM volume between HCs and patients with glioma in the left temporal lobe before surgery. (a) The two‐sample *t* test map between HCs and patients with the TG.R mask after controlling effects for age, gender, education, and TIV FDR (corrected *p*‐value of <.001). (b) (i) The two‐sample *t* test of GM volume between groups with the significantly increased brain areas in patients with glioma in the right temporal gyrus (left, yellow); (ii) the two‐sample *t* test of GM volume between groups in the right superior occipital gyrus (middle, red); (iii) the two‐sample *t* test of GM volume between groups in the right thalamus (right, green). Abbreviations: FDR, false discovery rate; GM, gray matter; HCs, healthy controls; ITG.R, right inferior temporal gyrus; SOG.R, right superior occipital gyrus; TG.R, right temporal gyrus; THA.R, right thalamus; TP Osup.R, right temporal pole (superior temporal gyrus)

**Figure 2:**
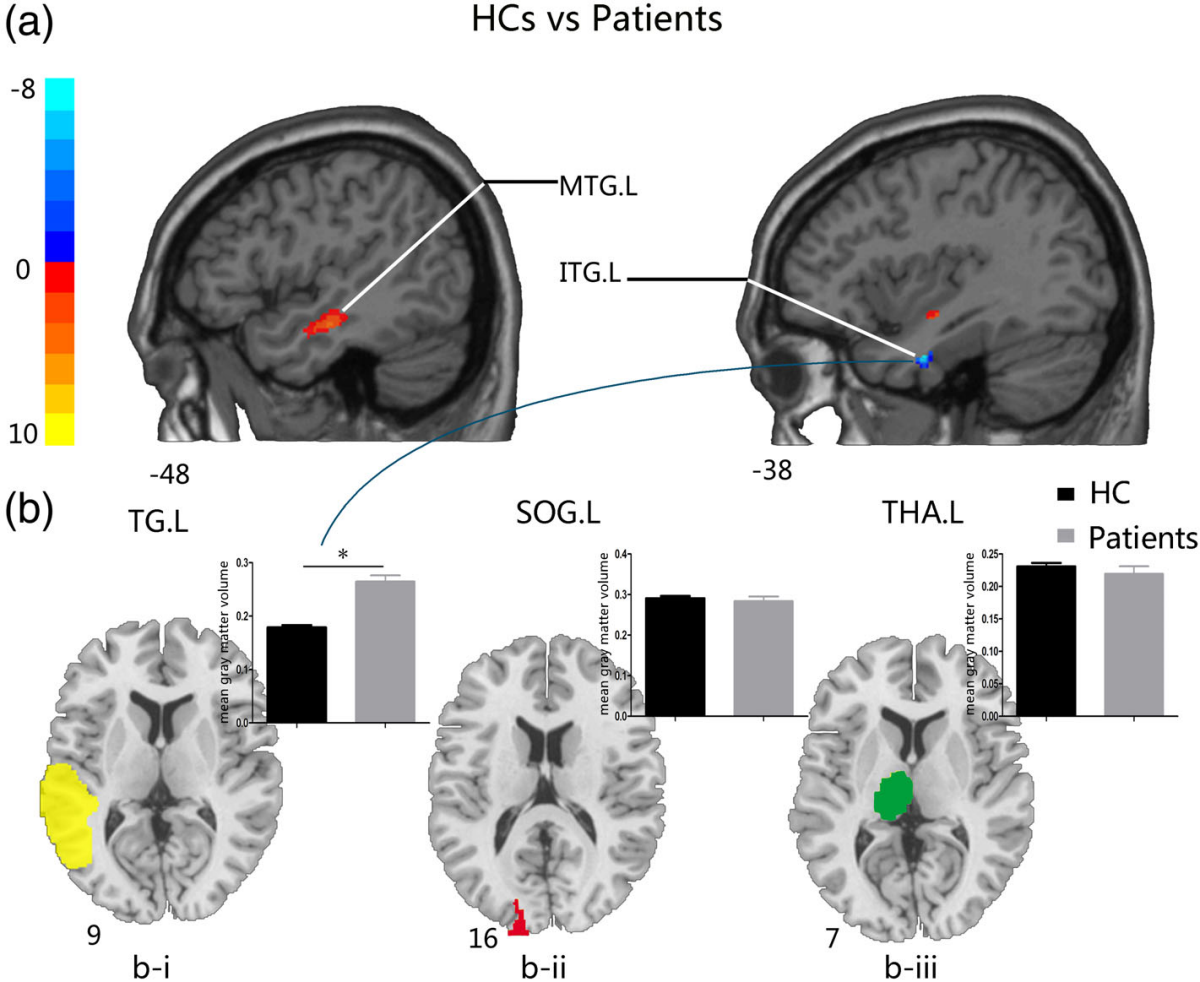
Comparisons of GM volume between HCs and patients with glioma in the right temporal lobe before surgery. (a) The two‐sample *t* test map between HCs and glioma patients with the TG.L mask after controlling effects for age, gender, education, and total intracranial volume (FDR corrected *p*‐value of <.001). (b) (i) The two‐sample *t* test of GM volume between groups on the significantly increased brain areas in patients with glioma in the left temporal gyrus (left, green); (ii) the two‐sample *t* test of GM volume between groups in the left superior occipital gyrus (middle, red); (iii) the two‐sample *t* test of GM volume between groups in the left thalamus (right, green). Abbreviations: FDR, false discovery rate; GM, gray matter; HCs, healthy controls; ITG.L, left inferior temporal gyrus; MTG.L, left middle temporal gyrus; SOG.L, left superior occipital gyrus; TG.L, left temporal gyrus; THA.L, left thalamus

**Figure 3:**
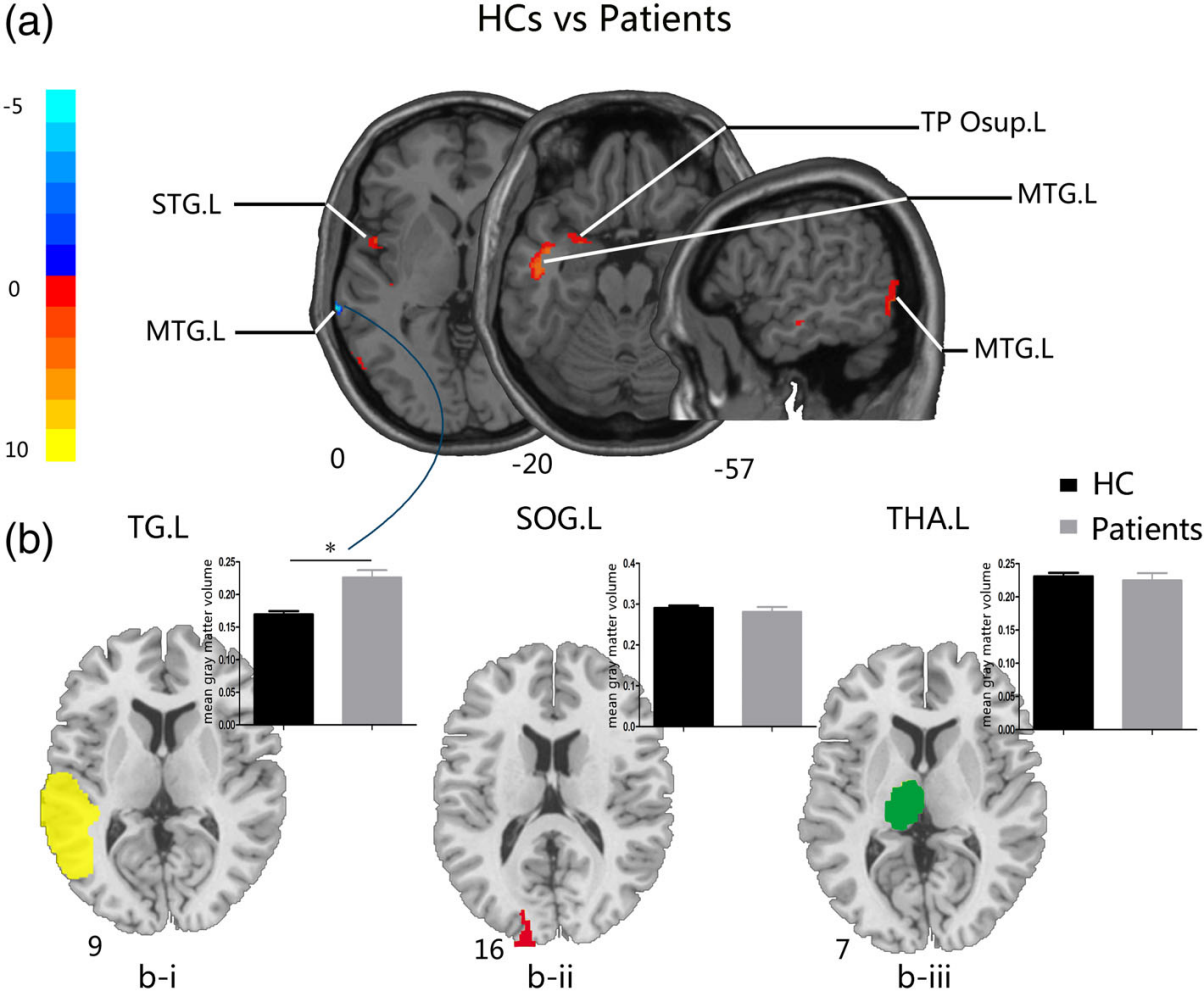
Comparisons of GM volume between HCs and patients with glioma in the right temporal lobe after surgery. (a) The two‐sample *t* test map between HCs and patients with the TG.L mask after controlling effects for age, gender, education, and total intracranial volume (FDR corrected *p* < .01). (b) (i) The two‐sample *t* test of GM volume between groups on the significantly increased brain areas in patients with glioma in the left temporal gyrus (left, yellow); (ii) the two‐sample *t* test of GM volume between groups in the left superior occipital gyrus (middle, red); (iii) the two‐sample *t* test of GM volume between groups in the left thalamus (right, green). Abbreviations: FDR, false discovery rate; HCs, healthy controls; GM, gray matter; TG.L, left temporal gyrus; SOG.L, left superior occipital gyrus; THA.L, left thalamus; TP Osup.L, left temporal pole (superior temporal gyrus); MTG.L, left middle temporal gyrus; STG.L, left superior temporal gyrus

We compared GM volume between patients with glioma and HCs by two‐sample *t* test before and after surgery. First, left and right temporal lobe region of interest (ROI) masks were created using the WFU‐Pickatlas toolbox implemented in MATLAB. Then, for patients with glioma in left temporal lobe before surgery, we compared the GM volume between HCs and left temporal glioma patients with the right temporal lobe ROI mask using REST toolbox with two‐sample *t* test (Figure 1). Similarly, we compared GM volume between HCs and patients with glioma in right temporal lobe, patients with glioma located in the left temporal lobe with ROI mask before surgery (Figure 2). To investigate the impact of surgery, we also compared GM volume between HCs and patients with glioma with ROI mask for the right or left temporal lobe after surgery (Figure 3), respectively. Apparently, to better understand the impact of surgery, we compared GM volume before surgery and after surgery in unilateral temporal glioma patients with two‐sample *t* test and paired *t* test with the contralateral temporal lobe ROI mask.

According to previous studies, we also selected some other control ROI region masks including left superior occipital gyrus (SOG.L), right SOG (SOG.R), left thalamus (THA.L), and right thalamus (THA.R) (Almairac et al., 2018) by WFU‐Pickatlas toolbox. Then, we compared GM volume between HCs and patients with the contralateral control ROI region masks before and after surgery with two‐sample *t* test, respectively. Furthermore, comparisons of GM volume between preoperative and postoperative patients were also performed with two‐sample *t* test and paired *t* test with the contralateral control ROI masks, respectively. All the control ROI masks were in the contralateral hemisphere, the SOG was far from the temporal lobe, and the thalamus was also relatively close to the lesion. Thus, by combing the temporal lobe ROI mask and the two control ROI masks, we could clearly identify whether the impact of the lesions restrictedly located in contralateral temporal lobe or may also influence the other far or close brain regions.

The cluster size >30 voxels were applied for multiple comparisons at the voxel level. All the two‐sample *t* tests were performed after correcting for the confounding factors including age, gender, education, and TIV with false discovery rate (FDR) corrected *p*‐value of <.001; besides, comparisons between preoperative and postoperative patients were corrected with FDR, *p* < .01 for age, gender, education, TIV, and tumor volume as covariates.

### 2.6 Statistical analysis

#### 2.6.1 Demographic and neurocognitive characteristics

**Figure 4:**
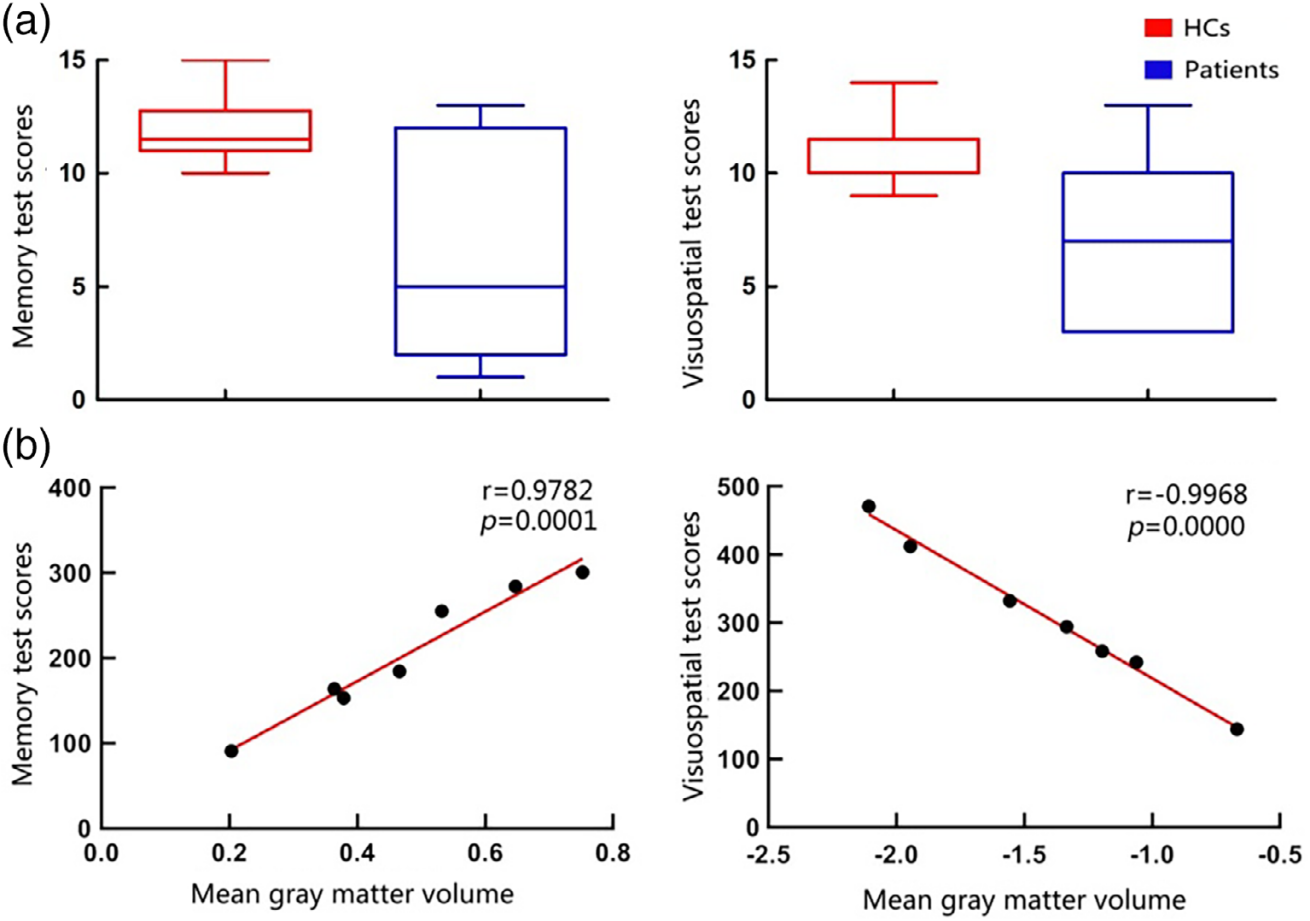
Comparison of cognitive scores between HCs and patients with glioma, and the relationship between mean GM volume in significantly increased brain areas and several cognitive functions in patients with glioma. (a) The comparison of cognitive scores between HCs and patients. There are no significant differences between groups following Bonferroni correction. (b) The relationship between mean GM volume of significantly increased brain areas and several cognitive functions in patients with glioma. The *p*‐values smaller than .007 (Bonferroni corrected *p*‐value of <.05/7) was considered statistically significant. Note. Due to some participants did not complete the cognitive scale assessment, there were seven subjects have scores of memory test (one left temporal glioma patient and six right temporal glioma patients) and visuospatial test (two left temporal glioma patients and five right temporal glioma patients), respectively. Abbreviations: HCs, healthy controls; GM, gray matter

We performed one‐way analysis of variance for the statistical analysis of the patients' demographics including age, TIV, and education among groups. And a *χ* ^2^ test was performed for sex ratio (Table 1). The two‐tailed two‐sample t test was performed to compare neurocognitive scores between HCs and glioma patients, with Bonferroni, corrected *p*‐value of *p* < .05/7 (Table 2, Figure 4a).

**Table 1:** Demographic characteristics of patients with gliomas and healthy control

	Patients		HCs	*p‐*Values
Variable	LTGP	RTGP		
No.	8	9	28	NA
Number of after surgery	3	6		
Age, year	57.25 ± 7.52 (47–70)	51.56 ± 17.56 (21–69)	56.82 ± 7.74 (42–70)	.385
Education, year	7.13 ± 3.91 (0–12)	8.33 ± 3.91 (0–12)	11.29 ± 3.53 (1–18)	.01*
Sex ratio, F/M, *n*	2/6	3/6	18/10	.072
Handedness	R	R	R	NA
Tumor volume, cm^3^	102.95 ± 65.61 (36.75–237.60)	105.25 ± 71.52 (36.75–246.51)	NA	NA
Total intracranial volume, cm^3^	1,440.85 ± 124.06 (1,272.84–1,611.80)	1,412.49 ± 110.84 (1,253.76–1,591.87)	1,395.69 ± 84.68 (1,248.16–1,570.98)	.51

*Note*. Data were expressed as the mean ± *SD* (minimum value–maximum value). *Significant differences were found between groups. The *p*‐values were determined using one‐way ANOVA for age, total intracranial volume, and education; however, a *χ* ^2^ test was used for sex ratio.

Abbreviations: ANOVA, analysis of variance; HCs, healthy controls; LTGP, left temporal glioma patients; NA, not applicable; R, right‐handed; RTGP, right temporal glioma patients.

**Table 2:** Cognitive scores of patients with gliomas and healthy controls

Scores of each cognitive domain	Patients	HCs	*p*‐Values
DST	8.44 ± 3.09	11.00 ± 2.67	.518
Memory test	6.43 ± 4.72	11.88 ± 1.55	.063
Visuospatial test	7.00 ± 3.87	10.63 ± 1.60	.021
Math exam	5.89 ± 2.42	10.63 ± 2.00	.007*
DSST	7.40 ± 2.88	11.88 ± 1.64	.028*
Mapping	5.63 ± 2.56	9.88 ± 0.64	.000*
Similarity	6.38 ± 2.88	10.00 ± 1.07	.035*

*Note*. Each cognition scores of patients with gliomas and HCs were expressed as the mean ± *SD*. The significant cognitive scores were obtained by two‐sample *t* tests and *p*‐values of less than .007 (Bonferroni corrected *p*‐value of <.05/7) was considered statistically significant, symbolized with *.

Abbreviations: DDST, Digital Symbol Substitution Test; DST, Digit Span Test; HCs, healthy controls.

#### 2.6.2 The clinical significance of altered GM volume in patients

To investigate the relationship between cognitive ability and increased GM volume, we performed correlation analysis between neurocognitive scores and the increased GM volume before surgery. However, due to some participants did not complete the cognitive scale assessment, there were few subjects have memory tests (one left temporal glioma patient and six right temporal glioma patients) and visuospatial tests (two left temporal glioma patients and five right temporal glioma patients). To ensure the veracity of the correlation analysis, we controlled the effect of age, gender, education, TIV, and tumor volume (using in‐house code running in MATLAB), followed by Bonferroni corrected *p*‐value of *p* < .05/7.

The Statistical Package for the Social Sciences (SPSS) for Windows, version 22 (SPSS, Chicago, IL) was used for all the statistical analysis.

## 3 RESULTS

### 3.1 Demographic and neurocognitive characteristics

As summarized in Table 1, no significant differences in age (*p* = .385), gender (*p* = .072), and TIV (*p* = .51) were observed among groups. However, a significant difference of the education (*p* = .01) between three groups (patients with glioma in left temporal lobe, patients with glioma located in right temporal lobe, and HCs) was observed. Compared to HCs, the scores of math exam, DSST, mapping, and similarity cognition of patients with glioma were significantly impaired. Nevertheless, scores of DST, memory, and visuospatial cognition did not show significant differences between groups (Table 2, Bonferroni corrected *p*‐value of *p* < .05/7).

### 3.2 Comparative analysis of GM volume

#### 3.2.1 Preoperative GM volume comparison between HCs and patients

For the patients with glioma in left temporal lobe group, increased GM volumes were observed in the right inferior temporal gyrus (ITG.R) and right temporal pole (superior temporal gyrus; TP Osup.R) (Table 3, Figure 1a). We also compared the mean GM volume between HCs and patients with glioma in the left temporal lobe in the two increased brain regions (Figure 1b‐i), and the patients with glioma in left temporal lobe exhibited significantly increased GM volume. However, we did not find significant difference between groups with the SOG.R and the THA.R masks (Figure 1b‐ii and ‐iii).

**Table 3:** Peak locations of regions: comparison of GM volume between patients with glioma and healthy controls

	Areas	Peak location			Cluster size	*T*
		(MNI coordinates)				
		*X Y Z*			(voxels)	
Before surgery	HCs vs. LTGP					
	ITG.R	60	−66	−12	53	−6.8356
	TP Osup.R	64.5	10.5	−3	173	−6.9332
	HCs vs. RTGP					
	ITG.L	−39	−4.5	−30	51	−8.377
	MTG.L	−25.5	3	−19.5	362	10.472
After surgery	HCs vs. LTGP					
	NA	NA	NA	NA	NA	NA
	HCs vs. RTGP					
	MTG.L	−43.5	−15	−12	882	11.3545
	TP Osup.L	−25.5	3	−19.5	46	5.4296
	MTG.L	−54	−69	−10.5	113	6.3304
	MTG.L	−72	−34.5	−2.8e‐14	32	−5.6541
	STG.L	−49.5	3	−2.8e‐14	32	5.3404

*Note*. Results before surgery were obtained after controlling effects of age, gender, education, and total intracranial volume (FDR corrected *p*‐value of <.001). Results after surgery were obtained after controlling effects of age, gender, education, and total intracranial volume (FDR corrected *p*‐value of <.01).

Abbreviations: FDR, false discovery rate; GM, gray matter; HCs, healthy controls; ITG.L, left inferior temporal gyrus; ITG.R, right inferior temporal gyrus; LTGP, left temporal glioma patients; MTG.L, left middle temporal gyrus; RTGP, right temporal glioma patients; TP Osup.R, right temporal pole (superior temporal gyrus); TP Osup.L, left temporal pole (superior temporal gyrus); STG.L, left superior temporal gyrus.

For the patients with glioma in right temporal lobe group, increased GM volume was observed in the left inferior temporal gyrus (ITG.L) and decreased GM volume was found in the left middle temporal gyrus (MTG.L) (Table 3, Figure 2a). We also compared the mean GM volume between HCs and patients within the increased brain region (Figure 2b‐i), and the patients with glioma in the right temporal lobe exhibited significantly increased GM volume. However, we did not find a significant difference between groups with the SOG.L and the THA.L masks (Figure 2b‐ii and ‐iii).

The above results were FDR corrected *p* value of <.001, cluster size >30 voxels.

#### 3.2.2 Postoperative GM volume comparison between HCs and patients

Compared to HCs, patients with glioma in left temporal lobe exhibited no significant difference with all masks. For the left temporal lobe ROI mask compared to HCs, patients with glioma in right temporal lobe showed significantly higher GM volume in the MTG.L and decreased GM volumes in the STG.L, in the left temporal pole (superior temporal gyrus; TP Osup.L), and the MTG.L (Table 3, Figure 3a). GM volume comparison between HCs and patients with glioma in the right temporal lobe in the increased brain region (Figure 3b‐i) showed that GM volume of patients with glioma in right temporal lobe was significantly higher. Similar to the preoperative results, there were no significant differences between groups in SOG.L and THA.L masks (Figure 3b‐ii and ‐iii). The above results were FDR corrected *p* value of <.01, cluster size >30 voxels.

#### 3.2.3 GM volume comparison between preoperation and postoperation

Next, to investigate the impacts of the tumor resection, we compared GM volume between preoperative and postoperative groups in the patients with glioma in left temporal lobe group and the patients with glioma in right temporal lobe group, respectively; however, there was no significant difference. The above results were FDR corrected *p* value of <.01, cluster size >30 voxels.

### 3.3 The clinical significance of altered GM volume in patients

Preoperatively, correlation analysis was performed on the seven cognitive abilities including DST, memory test, visuospatial test, math exam test, DSST, mapping test, and similarity test. The memory test was significantly positively correlated with the increased GM volume (*r* = .9782; *p* = .0001), and the visuospatial test was significantly negatively correlated with the increased GM volume (*r* = −.9968; *p* = .0000) (Figure 4). However, DST, math exam test, DSST, mapping test, and similarity test did not show any significant correlation with increased GM volume in the contralateral temporal lobe of glioma patients.

## 4 DISCUSSION

The present study investigated the restructuring of contralateral GM volume before and after surgery and the correlation between these alterations in contralateral GM volume with cognitive functions in patients with unilateral temporal lobe glioma. Consistent with our hypothesis, this study demonstrated that there was a compensatory increase in the contralateral temporal GM volume in patients with unilateral temporal lobe glioma. And the altered GM volume was significantly correlated with some of the cognitive functions, which possibly was attributed to compensatory mechanism in brain. However, we also found moderate changes in GM volume postoperatively in the contralateral temporal lobe compared to the preoperative alteration. Taken together, these findings indicated a compensatory mechanism to brain injury in patients with temporal glioma.

Furthermore, this study also revealed that the effects of the tumor were not limited to the region of lesion, but were more likely to elicit structural and connectivity alteration in distant brain areas, expanding even to the opposite hemisphere of the brain (Almairac et al., 2018; Bouwen et al., 2018; Hart et al., 2019; Maesawa et al., 2015). Consistent with a previous study, the present study also indicated identical consequence of increased GM volume in the contralateral temporal lobe (Almairac et al., 2018; Xu et al., 2017). For patients with glioma located in left temporal lobe before surgery, we found that two brain areas with increased GM volume with the ITG.R and TP Osup.R of the right temporal lobe ROI mask compared with HCs. However, for the patients with glioma located in right temporal lobe before surgery, increased GM volume in ITG.L was recorded; however, decrease in the MTG.L was observed in the left temporal lobe ROI mask compared with HCs. Previous studies reported that ITG is associated with mathematical processing (Grotheer, Jeska, & Grill‐Spector, 2018) and visual perception (Onitsuka et al., 2004), and temporal pole is associated with various higher order socioemotional cognition functions; however, the exact function remains elusive (Pehrs et al., 2017). Though the GM volume in contralateral ITG and TP Osup.R was increased, the adverse effect of the lesion may be more prominent; as reflected by math exam test scores of patients, which was relatively lower than that of HCs. However, with no apparent increase in GM volume, the cognitive function of patients might have been worse. It was worth noting that the patients with glioma in left temporal lobe and right temporal lobe both exhibited a similar increased brain area (ITG) which suggested that ITG may be an essential compensatory brain area in patients with temporal glioma. The MTG is involved in several cognitive abilities including language and semantic memory processing (Onitsuka et al., 2004). The decreased GM volume located in the MTG.L may lead to decrease in memory test scores; however, in this study, the patients with glioma only exhibited a decreasing trend. This may be attributed to the robustness of the brain function; more precisely, the decrease in GM volume in MTG was not adequate enough to cause a decline in patients' neurocognitive function. Thus, in order to confirm our hypothesis and further examine the compensatory mechanism, we compared the two controlling ROI masks (SOG and thalamus); however, no significant difference was found in GM volume changes on applying these masks in patients with glioma. Collectively, this study indicated that the compensatory increase in GM volume may only occur in the contralateral homotopic temporal lobe in patients with unilateral temporal lobe glioma.

Although the preoperative data analysis demonstrated a possible compensatory mechanism, we also conducted postoperative data analysis; however, the postoperative patients with glioma exhibited partial loss in compensatory mechanism compared with the preoperative findings. This may be attributed to the fact that only three MRI data were available for patients with glioma in left temporal lobe after surgery; thus, comparison between HCs and patients with left temporal glioma after surgery was inconclusive. Thus, further studies on large sample size are warranted to confirm these findings in patients with glioma before surgery. On comparison between HCs and patients with glioma located in right temporal lobe, with increase or decrease in brain regions, no significant difference was observed with left temporal ROI mask. Even with data on only six patients with right temporal glioma after surgery, the brain areas with alterations after surgery elicited certain changes compared to that before surgery, suggesting a possible compensatory mechanism prevailing in the contralateral temporal lobe. Three regions with significant alterations were located in the MTG.L; of these, GM volume was decreased in two whereas increased in one. Furthermore, decreased GM volume in the TP Osup.L and STG.L was observed, in which the STG was markedly related to auditory word‐form recognition (DeWitt & Rauschecker, 2013; DeWitt & Rauschecker, 2016), and maybe a fundamental of neurobiology in obsessive–compulsive disorder (Fan et al., 2017). Due to the postoperative brain structural alterations and the influence of CSF, the brain region with altered GM volume may be slightly different from that of the preoperative, thus, an increase in GM volume in the contralateral homotopic temporal lobe was observed.

Although preoperative comparison and postoperative comparison exhibited some relative differences, we did not find significant differences between preoperative and postoperative findings using two‐sample *t* test or paired *t* test. This phenomenon indicated that neurons exhibited robustness in its ability to maintain balance between stability and flexibility even in changing environments (Hiesinger & Hassan, 2018).

Previous studies reported that gliomas may significantly impair cognitive functions (Derks et al., 2014; Liu et al., 2019; Noll et al., 2015; Pace et al., 2017; van Kessel, Baumfalk, van Zandvoort, Robe, & Snijders, 2017). Notably, significant differences were found in math exam test, DSST, mapping test, and similarity test between HCs and in patients with glioma before surgery, which was partially consistent with previous studies. The cognitive deficit may be a gradual process, to avoid cognitive function from deteriorating or obtain better outcomes; surgery might be the most suitable treatment of care (Satoer et al., 2014). Accumulating studies have suggested that temporal lobe is associated with visuospatial function (Noll et al., 2015; Whittingstall, Bernier, Houde, Fortin, & Descoteaux, 2014). In the present study, visuospatial test score was negatively correlated with increased GM volume in the contralateral temporal lobe. Even there was no significant difference in visuospatial test scores between HCs and patients; however, patients still exhibited a declining trend. Although increased GM volume compensates for some of the function, it may not be adequate enough to offset the cognitive impairment associated with temporal lobe injury, suggesting that increased GM volume may imply more severe progression of glioma. The previous study demonstrated that temporal lobe is also involved in the function of memory (Onitsuka et al., 2004). In the present study, we found that memory test score was positively correlated with increased GM volume in the contralateral temporal lobe. Moreover, memory test comparison between HCs and glioma patients exhibited no significant difference. With the progressing glioma, the scores of the memory test should decrease; however, we did not find any difference in this study, suggesting that the increased GM volume in the contralateral temporal lobe compensates for the damage caused by the lesion. Overall, increased GM volume in glioma patients indicates high compensation for brain function; however, also implicate more severe illness.

## 5 LIMITATION

Although this study was carefully designed, there were still some limitations to our study. First, the study was limited by a small number sample size, which may lead to unrepresentative results. Additionally, the number of patients after surgery is also small. To compensate for this, we applied two additional controlling masks and performed the same analysis in patients with left or right temporal lobe, respectively. We will continue to enroll patients for our future studies and would analyze the data on a larger sample size to improve the reliability of the results. Another limitation is that significant difference in education was presented among groups which may influence the accuracy of our results. To reduce the impact of education, we performed analysis with education as a covariate. For the nonsignificant demographics data, we analyzed them as covariates for further analysis. Thus, our results are still reliable. Finally, we only analyzed the correlation between preoperative cognitive function and GM due to the loss of data on cognitive function postoperatively. We will strengthen data management and perform postoperative cognitive function analysis in our future studies.

## 6 CONCLUSION

The compensatory increase in GM volume in the patients with unilateral temporal lobe occurs only in the contralateral temporal lobe, which still exists even after the resection of tumor. Furthermore, the correlation between cognition functions (memory and visuospatial tests) and increased GM volume suggested that the increased GM in the contralateral temporal lobe is a functional compensatory mechanism. These findings indicated that more the increase in GM volume, more the compensation for brain function; however, it also imply more severe illness homologous to lesioned tissue owing to the heterogeneous nature of the temporal lobe. Collectively, this study provides an important new perspective for the neurosurgical evaluation and better management of glioma and the preservation of cognitive function.

## CONFLICT OF INTEREST

The authors declare that they have no conflict of interest.

## AUTHOR CONTRIBUTIONS

Conceived and designed the experiments: H.L., J.C., X.H. Preprocessed and analyzed MRI data: D.L., J.C., X.H. Contributed materials/analysis tools: K.Y., C.X., C.X. Preparation of the article, figures, and tables: D.L., G.H., J.C., X.H., Y.L., Y.Z. All authors read, revised, and approved the final version of the manuscript.

## Data Availability

The data of subjects are not publicly available due to privacy or ethical restrictions.

## References

[hbm24911-bib-0001] Almairac, F. , Duffau, H. , & Herbet, G. (2018). Contralesional macrostructural plasticity of the insular cortex in patients with glioma. Neurology, 91(20), e1902–e1908. 30305447 10.1212/WNL.0000000000006517

[hbm24911-bib-0002] Ashburner, J. , & Friston, K. J. (2000). Voxel‐based morphometry—The methods. NeuroImage, 11(6 Pt. 1), 805–821. 10860804 10.1006/nimg.2000.0582

[hbm24911-bib-0003] Ashburner, J. , & Friston, K. J. (2009). Computing average shaped tissue probability templates. NeuroImage, 45(2), 333–341. 19146961 10.1016/j.neuroimage.2008.12.008

[hbm24911-bib-0004] Bouwen, B. L. J. , Pieterman, K. J. , Smits, M. , Dirven, C. M. F. , Gao, Z. , & Vincent, A. (2018). The impacts of tumor and tumor associated epilepsy on subcortical brain structures and long distance connectivity in patients with low grade glioma. Frontiers in Neurology, 9, 1004. 30538668 10.3389/fneur.2018.01004 PMC6277571

[hbm24911-bib-0005] Dang, C. , Liu, G. , Xing, S. , Xie, C. , Peng, K. , Li, C. , … Zeng, J. (2013). Longitudinal cortical volume changes correlate with motor recovery in patients after acute local subcortical infarction. Stroke, 44(10), 2795–2801. 23929747 10.1161/STROKEAHA.113.000971

[hbm24911-bib-0006] Derks, J. , Reijneveld, J. C. , & Douw, L. (2014). Neural network alterations underlie cognitive deficits in brain tumor patients. Current Opinion in Oncology, 26(6), 627–633. 25188475 10.1097/CCO.0000000000000126

[hbm24911-bib-0007] DeWitt, I. , & Rauschecker, J. P. (2013). Wernicke's area revisited: Parallel streams and word processing. Brain and Language, 127(2), 181–191. 24404576 10.1016/j.bandl.2013.09.014 PMC4098851

[hbm24911-bib-0008] DeWitt, I. , & Rauschecker, J. P. (2016). Convergent evidence for the causal involvement of anterior superior temporal gyrus in auditory single‐word comprehension. Cortex, 77, 164–166. 26387007 10.1016/j.cortex.2015.08.016 PMC7177176

[hbm24911-bib-0009] Duffau, H. (2005). Lessons from brain mapping in surgery for low‐grade glioma: Insights into associations between tumour and brain plasticity. The Lancet Neurology, 4(8), 476–486. 16033690 10.1016/S1474-4422(05)70140-X

[hbm24911-bib-0010] Duffau, H. (2006). New concepts in surgery of WHO grade II gliomas: Functional brain mapping, connectionism and plasticity—A review. Journal of Neuro‐Oncology, 79(1), 77–115. 16607477 10.1007/s11060-005-9109-6

[hbm24911-bib-0011] Duffau, H. (2014). The huge plastic potential of adult brain and the role of connectomics: New insights provided by serial mappings in glioma surgery. Cortex, 58, 325–337. 24050218 10.1016/j.cortex.2013.08.005

[hbm24911-bib-0012] Duffau, H. , & Taillandier, L. (2015). New concepts in the management of diffuse low‐grade glioma: Proposal of a multistage and individualized therapeutic approach. Neuro‐Oncology, 17(3), 332–342. 25087230 10.1093/neuonc/nou153 PMC4483091

[hbm24911-bib-0013] Fan, J. , Zhong, M. , Gan, J. , Liu, W. , Niu, C. , Liao, H. , … Zhu, X. (2017). Spontaneous neural activity in the right superior temporal gyrus and left middle temporal gyrus is associated with insight level in obsessive‐compulsive disorder. Journal of Affective Disorders, 207, 203–211. 27723545 10.1016/j.jad.2016.08.027

[hbm24911-bib-0014] Fauth, M. , & Tetzlaff, C. (2016). Opposing effects of neuronal activity on structural plasticity. Frontiers in Neuroanatomy, 10, 75. 27445713 10.3389/fnana.2016.00075 PMC4923203

[hbm24911-bib-0015] Ghinda, D. C. , Wu, J. S. , Duncan, N. W. , & Northoff, G. (2018). How much is enough—Can resting state fMRI provide a demarcation for neurosurgical resection in glioma? Neuroscience and Biobehavioral Reviews, 84, 245–261. 29198588 10.1016/j.neubiorev.2017.11.019

[hbm24911-bib-0016] Grotheer, M. , Jeska, B. , & Grill‐Spector, K. (2018). A preference for mathematical processing outweighs the selectivity for Arabic numbers in the inferior temporal gyrus. NeuroImage, 175, 188–200. 29604456 10.1016/j.neuroimage.2018.03.064 PMC6173953

[hbm24911-bib-0017] Gusyatiner, O. , & Hegi, M. E. (2018). Glioma epigenetics: From subclassification to novel treatment options. Seminars in Cancer Biology, 51, 50–58. 29170066 10.1016/j.semcancer.2017.11.010

[hbm24911-bib-0018] Hart, M. G. , Romero‐Garcia, R. , Price, S. J. , & Suckling, J. (2019). Global effects of focal brain tumors on functional complexity and network robustness: A prospective cohort study. Neurosurgery, 84(6), 1201–1213. 30137556 10.1093/neuros/nyy378 PMC6520100

[hbm24911-bib-0019] Hiesinger, P. R. , & Hassan, B. A. (2018). The evolution of variability and robustness in neural development. Trends in Neurosciences, 41(9), 577–586. 29880259 10.1016/j.tins.2018.05.007

[hbm24911-bib-0020] Kane, J. R. (2015). From histology to neurocognition: The influence of tumor grade in glioma of the left temporal lobe on neurocognitive function. Neuro‐Oncology, 17(10), 1420–1421. 26395062 10.1093/neuonc/nov150 PMC4578591

[hbm24911-bib-0021] Liu, C. H. , Jing, B. , Ma, X. , Xu, P. F. , Zhang, Y. , Li, F. , … Wang, C. Y. (2014). Voxel‐based morphometry study of the insular cortex in female patients with current and remitted depression. Neuroscience, 262, 190–199. 24406440 10.1016/j.neuroscience.2013.12.058

[hbm24911-bib-0022] Liu, D. , Hu, X. , Liu, Y. , Yang, K. , Xiao, C. , Hu, J. , … Liu, H. (2019). Potential intra‐ or cross‐network functional reorganization of the triple unifying networks in patients with frontal glioma. World Neurosurgery, 128, e732–e743. 31077892 10.1016/j.wneu.2019.04.248

[hbm24911-bib-0023] Louis, D. N. , Perry, A. , Reifenberger, G. , von Deimling, A. , Figarella‐Branger, D. , Cavenee, W. K. , … Ellison, D. W. (2016). The 2016 World Health Organization classification of tumors of the central nervous system: A summary. Acta Neuropathologica, 131(6), 803–820. 27157931 10.1007/s00401-016-1545-1

[hbm24911-bib-0024] Maesawa, S. , Bagarinao, E. , Fujii, M. , Futamura, M. , Motomura, K. , Watanabe, H. , … Wakabayashi, T. (2015). Evaluation of resting state networks in patients with gliomas: Connectivity changes in the unaffected side and its relation to cognitive function. PLoS One, 10(2), e0118072. 25659130 10.1371/journal.pone.0118072 PMC4319851

[hbm24911-bib-0025] Matsuda, H. (2016). MRI morphometry in Alzheimer's disease. Ageing Research Reviews, 30, 17–24. 26812213 10.1016/j.arr.2016.01.003

[hbm24911-bib-0026] Noll, K. R. , Bradshaw, M. E. , Weinberg, J. S. , & Wefel, J. S. (2017). Relationships between neurocognitive functioning, mood, and quality of life in patients with temporal lobe glioma. Psychooncology, 26(5), 617–624. 26677053 10.1002/pon.4046 PMC4995149

[hbm24911-bib-0027] Noll, K. R. , Weinberg, J. S. , Ziu, M. , Benveniste, R. J. , Suki, D. , & Wefel, J. S. (2015). Neurocognitive changes associated with surgical resection of left and right temporal lobe glioma. Neurosurgery, 77(5), 777–785. 26317672 10.1227/NEU.0000000000000987 PMC4831270

[hbm24911-bib-0028] Noll, K. R. , Ziu, M. , Weinberg, J. S. , & Wefel, J. S. (2016). Neurocognitive functioning in patients with glioma of the left and right temporal lobes. Journal of Neuro‐Oncology, 128(2), 323–331. 27022915 10.1007/s11060-016-2114-0 PMC4884162

[hbm24911-bib-0029] Onitsuka, T. , Shenton, M. E. , Salisbury, D. F. , Dickey, C. C. , Kasai, K. , Toner, S. K. , … McCarley, R. W. (2004). Middle and inferior temporal gyrus gray matter volume abnormalities in chronic schizophrenia: An MRI study. The American Journal of Psychiatry, 161(9), 1603–1611. 15337650 10.1176/appi.ajp.161.9.1603 PMC2793337

[hbm24911-bib-0030] Ostrom, Q. T. , Gittleman, H. , Xu, J. , Kromer, C. , Wolinsky, Y. , Kruchko, C. , & Barnholtz‐Sloan, J. S. (2016). CBTRUS statistical report: Primary brain and other central nervous system tumors diagnosed in the United States in 2009–2013. Neuro‐Oncology, 18(Suppl. 5), v1–v75. 28475809 10.1093/neuonc/now207 PMC8483569

[hbm24911-bib-0031] Pace, A. , Dirven, L. , Koekkoek, J. A. F. , Golla, H. , Fleming, J. , Rudà, R. , … Taphoorn, M. J. B. (2017). European Association for Neuro‐Oncology (EANO) guidelines for palliative care in adults with glioma. The Lancet Oncology, 18(6), e330–e340. 28593859 10.1016/S1470-2045(17)30345-5

[hbm24911-bib-0032] Park, J. E. , Kim, H. S. , Kim, S. J. , Kim, J. H. , & Shim, W. H. (2015). Alteration of long‐distance functional connectivity and network topology in patients with supratentorial gliomas. Neuroradiology, 58(3), 311–320. 26635295 10.1007/s00234-015-1621-6

[hbm24911-bib-0033] Pehrs, C. , Zaki, J. , Schlochtermeier, L. H. , Jacobs, A. M. , Kuchinke, L. , & Koelsch, S. (2017). The temporal pole top‐down modulates the ventral visual stream during social cognition. Cerebral Cortex, 27(1), 777–792. 26604273 10.1093/cercor/bhv226

[hbm24911-bib-0034] Satoer, D. , Visch‐Brink, E. , Smits, M. , Kloet, A. , Looman, C. , Dirven, C. , & Vincent, A. (2014). Long‐term evaluation of cognition after glioma surgery in eloquent areas. Journal of Neuro‐Oncology, 116(1), 153–160. 24173681 10.1007/s11060-013-1275-3

[hbm24911-bib-0035] van Kessel, E. , Baumfalk, A. E. , van Zandvoort, M. J. E. , Robe, P. A. , & Snijders, T. J. (2017). Tumor‐related neurocognitive dysfunction in patients with diffuse glioma: A systematic review of neurocognitive functioning prior to anti‐tumor treatment. Journal of Neuro‐Oncology, 134(1), 9–18. 28567586 10.1007/s11060-017-2503-z PMC5543199

[hbm24911-bib-0036] Vanasse, T. J. , Fox, P. M. , Barron, D. S. , Robertson, M. , Eickhoff, S. B. , Lancaster, J. L. , & Fox, P. T. (2018). BrainMap VBM: An environment for structural meta‐analysis. Human Brain Mapping, 39(8), 3308–3325. 29717540 10.1002/hbm.24078 PMC6866579

[hbm24911-bib-0037] von Bernhardi, R. , Bernhardi, L. E. , & Eugenin, J. (2017). What is neural plasticity? Advances in Experimental Medicine and Biology, 1015, 1–15. 29080018 10.1007/978-3-319-62817-2_1

[hbm24911-bib-0038] Voytek, B. , Davis, M. , Yago, E. , Barcelo, F. , Vogel, E. K. , & Knight, R. T. (2010). Dynamic neuroplasticity after human prefrontal cortex damage. Neuron, 68(3), 401–408. 21040843 10.1016/j.neuron.2010.09.018 PMC3005706

[hbm24911-bib-0039] Whittingstall, K. , Bernier, M. , Houde, J. C. , Fortin, D. , & Descoteaux, M. (2014). Structural network underlying visuospatial imagery in humans. Cortex, 56, 85–98. 23514930 10.1016/j.cortex.2013.02.004

[hbm24911-bib-0040] Wu, A. S. , Witgert, M. E. , Lang, F. F. , Xiao, L. , Bekele, B. N. , Meyers, C. A. , … Wefel, J. S. (2011). Neurocognitive function before and after surgery for insular gliomas. Journal of Neurosurgery, 115(6), 1115–1125. 21905800 10.3171/2011.8.JNS11488

[hbm24911-bib-0041] Xu, J. , Elazab, A. , Liang, J. , Jia, F. , Zheng, H. , Wang, W. , … Hu, Q. (2017). Cortical and subcortical structural plasticity associated with the glioma volumes in patients with cerebral gliomas revealed by surface‐based morphometry. Frontiers in Neurology, 8, 266. 28649229 10.3389/fneur.2017.00266 PMC5465275

[hbm24911-bib-0042] Yan, C. G. , Wang, X. D. , Zuo, X. N. , & Zang, Y. F. (2016). DPABI: Data processing & analysis for (resting‐state) brain imaging. Neuroinformatics, 14(3), 339–351. 27075850 10.1007/s12021-016-9299-4

[hbm24911-bib-0043] Zhang, H. , Shi, Y. , Yao, C. , Tang, W. , Yao, D. , Zhang, C. , … Song, Z. (2016). Alteration of the intra‐ and cross‐ hemisphere posterior default mode network in frontal lobe glioma patients. Scientific Reports, 6, 26972. 27248706 10.1038/srep26972 PMC4888650

